# A Web-Based Contraception Decision Tool for Individuals With Health Conditions in US Outpatient Clinics: Protocol for a Mixed Methods Cluster Randomized Controlled Trial

**DOI:** 10.2196/71101

**Published:** 2025-12-29

**Authors:** Beatrice Palazzolo, James E Aikens, Ananda Sen, Timothy C Guetterman, Lorraine R Buis, Vanessa K Dalton, Brian J Zikmund-Fisher, Natabhona Mabachi, Minji Kang, Christine Dehlendorf, Murphy Van Sparrentak, Cory B Lutgen, Cynthia Wynn, Justine P Wu

**Affiliations:** 1 Department of Internal Medicine University of Michigan Medical School Ann Arbor, MI United States; 2 Department of Family Medicine University of Michigan Medical School Ann Arbor, MI United States; 3 Department of Obstetrics and Gynecology University of Michigan Medical School Ann Arbor, MI United States; 4 Department of Health Behavior and Health Equity School of Public Health University of Michigan Ann Arbor, MI United States; 5 DARTNet Institute Aurora, CO United States; 6 Department of Family and Community Medicine School of Medicine University of California, San Francisco San Francisco, CA United States

**Keywords:** contraception, decision making, multiple chronic conditions, chronic disease randomized controlled trial, cluster analysis

## Abstract

**Background:**

Choosing contraception is a highly personal, often complex decision. People with acute and chronic health conditions (eg, pulmonary embolism, diabetes, and hypertension) must deliberate additional factors including whether and to what extent a contraceptive method may affect their health conditions or interact with their medications. “My Health My Choice” (web application developed by Alfa Jango) is a web-based decision support tool designed to help patients understand their contraceptive options considering their health conditions, medications, and personal priorities.

**Objective:**

We will evaluate the efficacy of “My Health My Choice” (MHMC) use among people with diverse health conditions who seek contraception in US outpatient clinics.

**Methods:**

This mixed methods cluster randomized controlled trial will compare MHMC and usual contraceptive care (intervention arm) to usual contraceptive care only (control arm). Fourteen clinics that specialize in primary care or obstetrics and gynecology will be enrolled, with 7 clinics in each arm. We will enroll 726 patients (363 in each group) who (1) are 18-49 years old, (2) are able to speak and read English, (3) are seeking contraception for pregnancy prevention, and (4) have at least 1 health condition among 60 eligible health conditions and characteristics (eg, cigarette smoking and postpartum state). Aim 1 (quantitative) is to assess the primary outcome of patient-reported contraceptive nonuse (yes or no) at 3 months. Aim 2 (mixed methods) starts with a quantitative summary of patient-reported contraceptive use by method type (eg, pills and shot) and corresponding Medical Eligibility Risk Category (Category 1, 2, 3, or 4) based on the Centers for Disease Control and Prevention (CDC) US Medical Eligibility Criteria (US MEC) Contraceptive Guidelines. We will conduct exit interviews of a subsample of approximately 30 patients and 30 clinicians to qualitatively understand how MHMC and other contextual factors influenced perceptions about contraceptive risk and contraceptive decisions. Aim 3 (quantitative) is to explore patient-reported contraceptive decisional conflict (measured by the Decisional Conflict Scale) and patient-reported quality of interaction with their contraceptive provider (measured by the Interpersonal Quality of Family Planning Scale) as mediators of the effects of MHMC on the primary outcome. This paper describes the study protocol per the SPIRIT (Standard Protocol Items: Recommendations for Intervention Trials) checklist.

**Results:**

The National Institute of Child Health and Human Development funded this study (R01HD110570, Principal Investigator JPW) in 2023. The study team is collaborating with the DARTNet Institute (Aurora, Colorado) to enroll and prepare health clinics for trial launch. Fourteen clinics have enrolled in the trial and are in various stages of study preparation and regulatory approval. We anticipate patient enrollment to run from October 2025 to September 2028.

**Conclusions:**

This is the first mixed methods cluster randomized controlled trial of MHMC*,* a novel web-based decision support tool for people with health conditions.

**Trial Registration:**

ClinicalTrials.gov NCT07075536; https://clinicaltrials.gov/study/NCT07075536

**International Registered Report Identifier (IRRID):**

DERR1-10.2196/71101

## Introduction

### Background and Rationale

Contraceptive care is an essential health service that allows people to achieve their reproductive goals, including the prevention of undesired pregnancy [1]. Selecting contraception is highly personal and, for many, an overwhelming decision due to the sheer number of potential contraceptive methods [2,3]. There are 18 contraceptive methods (hereafter referred to as “methods”) [4] that include nonprescription methods, prescription methods, contraceptive devices, and permanent contraception (tubal surgeries and vasectomy). Contraceptive methods can be short-acting (taken daily or weekly), long-acting (effective for years), or permanent [4]. Each method has a unique benefit and side effect profile (eg, headache and irregular bleeding) that affects the user’s experience and likelihood of method continuation [4]. Contraceptive decision tools help patients understand the full range of methods and weigh their respective pros and cons in the context of their personal values and preferences [5].

People with acute and chronic health conditions, a growing proportion of contraceptive users, face additional and unique information needs [6]. First, they should understand the impact of a specific method on their health conditions; that is, whether a method improves, worsens, or has no impact on disease state or progression. Second, for those who would continue an unplanned pregnancy to childbirth, they should be informed about potential pregnancy-related risks, or lack thereof, in the context of their health conditions. Third, patients should be aware if their medications interact with hormonal contraception [7] or cause birth defects (eg, phenytoin) [8]. Ideally, health care providers should help process this information so that patients can weigh the pros and cons of different methods and select the best method for themselves.

However, studies have shown that people with health conditions inconsistently receive counseling to make informed contraceptive decisions [9-12]. For example, in outpatient settings, 10%-39% of patients report using combined hormonal contraception (CHC) despite having conditions that may preclude the safe use of CHCs [9,11,12]. Patients who cannot use CHCs should be fully informed about non-CHC methods based on their personal needs and preferences.

It is also important to bear in mind that most people with health conditions are medically eligible to use most methods [13]; that is, the proven or theoretical benefits of using a specific method generally outweigh any risks in the majority of cases. However, fear of side effects and health concerns are among the most common reasons cited for not using the most preferred method or not using any method among US contraceptive candidates in a nationally representative survey [14]. Contraceptive nonuse, defined as not using any method despite wanting to prevent pregnancy, is associated with the highest risk of unplanned pregnancy [15]. The use of any method reduces the risk of pregnancy by 70%-99.9%, depending on the method type [15]. Patients should receive accurate and balanced information about contraceptive-related health risks, including cases where there is no known risk, to prevent unnecessary avoidance of otherwise safe methods.

In prior randomized controlled trials (RCTs), contraceptive decision tools have been associated with improved patient contraceptive knowledge [16,17], improved quality of contraceptive care experience [16], reduced contraceptive decisional conflict [16], or increased use of prescription contraception [17,18], while others showed no significant differences in outcomes [19-21]. These trials primarily enrolled healthy young adults or adolescents [16,19,20] and did not address clinician knowledge regarding contraceptive use for patients with health conditions.

However, evidence-based clinical guidelines for contraceptive use by people with health conditions do exist. The Centers for Disease Control and Prevention (CDC) United States Medical Eligibility Criteria (US MEC) summarizes evidence-based and expert-informed recommendations for the safe use of contraceptive methods in the context of more than 60 health conditions and characteristics [13]. To distill the 124-page document for clinical use, the US MEC content is available as a free smartphone app and a downloadable color-coded PDF chart summary [22,23]. However, studies show that the US MEC is underused by clinicians, particularly in primary care [24-27] settings. A decision tool that engages both patients and their clinicians is a novel yet unexplored approach to promote informed contraceptive decision-making and use in the context of health conditions.

To address this unmet need, we designed and piloted a novel, theory-informed web-based tool called “My Health, My Choice” (MHMC) with technical support from Alfa Jango, a software design team [28]. MHMC is an interactive tool with a patient version and a clinician version. The patient version provides contraceptive education specific to a patient’s health history and contraceptive priorities; an interactive tool that groups methods by safety (per their reported health conditions and medications) and allows for side-by-side comparisons, and a Birth Control Summary that captures the patient’s contraceptive preferences and questions for their clinician. Using the clinician version, clinicians can review the patient’s Birth Control Summary and clinical decision support specific to that patient and harmonized with the US MEC Guidelines for individuals with health conditions and relevant characteristics (eg, smoking status and lactation status) [7]. We posit that MHMC will simultaneously address the information needs of patients and clinicians and facilitate interactive discussions, leading to the use of methods that are patient-preferred and safe. This paper details the study protocol for a cluster RCT to evaluate the efficacy of MHMC in 14 outpatient clinics in the United States that provide contraceptive services.

### Research Aims and Hypotheses

#### Quantitative Aims and Hypotheses

The primary aim is to quantitatively assess patient-reported contraceptive nonuse (yes or no) at 3 months (primary outcome).

- Hypothesis 1: Contraceptive nonuse will be less likely in the intervention arm than the control arm at 3 months.

The secondary aim is to quantitatively assess the Medical Eligibility Risk category for patient-reported contraceptive method use per the US MEC schema (Category 1=no risk; Category 2=Advantages generally outweigh theoretical or proven risks; Category 3=Theoretical or proven risks usually outweigh advantages; Category 4=unacceptable health risks) [7]. Clinically translated, Categories 1 and 2 indicate methods that are safe to use, Category 3 indicates methods that should be avoided unless more appropriate methods are not available or acceptable to the patient, and Category 4 indicates methods that should be avoided [7]. We will explore the potential impact of the intervention on contraceptive decisions across US MEC categories using qualitative and mixed methods analysis, with a focus on Category 3 cases.

- Hypothesis 2: Given the need for a careful and informed discussion to weigh the benefits and risks of Category 3 use, we hypothesize that the decision tool will facilitate this process, leading to more Category 3 use cases in the intervention arm than the control arm.

The exploratory aim is to quantitatively explore contraception decisional conflict measured via a modified Decisional Conflict Scale (DCS) [29], and the patient-reported quality of interaction with their contraceptive provider, as measured via the Interpersonal Quality of Family Planning Scale (IQFP) [30], as mediators of the effects of MHMC on the primary outcome.

- Hypothesis 3: The impact of MHMC on contraceptive nonuse will be mediated by lower DCS scores and higher IQFP scores.

#### Anticipated Qualitative and Mixed Methods Findings

Patients in the intervention arm, particularly those who report Category 3 contraceptive use, will describe a more nuanced understanding of contraceptive risks weighed against the risks of unplanned pregnancy than those in the control arm.

## Methods

### Trial Design and Rationale: A Cluster RCT With a Mixed Methods Design

**Figure 1:**
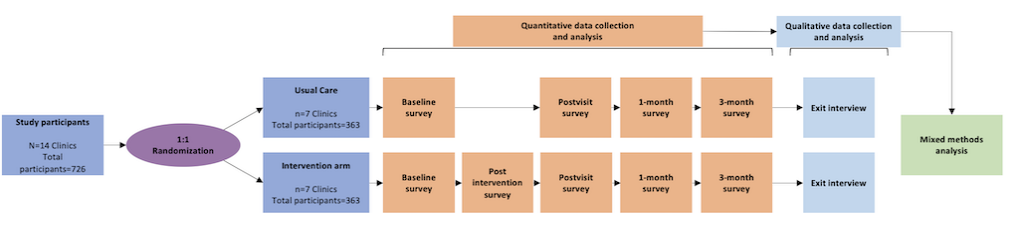
Mixed methods cluster RCT study design.

This study protocol reflects the SPIRIT (Standard Protocol Items: Recommendations for Intervention Trials) 2025 checklist for reporting RCTs [31]. For this efficacy trial, we will conduct a 2-arm, parallel cluster RCT using mixed methods to compare MHMC and usual care (intervention arm) to usual contraceptive care (control arm) in outpatient primary care or obstetrics and gynecology clinics in the United States. We will randomize at the level of the clinic site (the cluster unit) rather than the individual patient [32]. This group-level randomization approach prevents clinicians from influencing each other’s contraceptive practices or changing their contraceptive practices after exposure to MHMC, which then impacts the treatment of subsequent patients [32]. The rationale for incorporating qualitative methods and mixed methods (the integration of quantitative data and qualitative data) in this RCT is to more deeply understand how patients and providers navigate contraceptive decisions in the context of health conditions [33]. In particular, we seek to focus on challenging cases in which there is a method that should be avoided except in special circumstances when there is no appropriate or patient-acceptable alternative. We will use an explanatory sequential design [34], in which qualitative exit interviews will be done after survey data collection to explain our quantitative findings. Figure 1 depicts the mixed methods RCT design and the activities of each data collection and analysis phase.

Patients with upcoming clinic visits with their contraceptive provider will be screened on the phone and enrolled in the study. During the screening call, patients must confirm that they are 18-49 years old, potentially fertile, and desire to discuss contraception at their upcoming visit (refer to “Methods” section for full eligibility criteria). All patients will complete a baseline survey and attend their clinic visit as scheduled. Patients in the intervention arm will receive a password-protected web login to use MHMC on a smartphone, tablet, or computer prior to their clinic visit. The primary outcome (contraceptive nonuse) will be assessed at 1 month and 3 months to allow time for patients to schedule procedures for contraceptive devices or permanent contraception (eg, tubal surgery and vasectomy). Qualitative exit interviews will be conducted among a subset of 30 patients and 30 clinicians with a focus on US MEC Category 3 cases. These cases represent “gray-zone” situations in which clinicians and patients should carefully weigh the benefits and risks prior to using a specific contraceptive method. Informed by the RE-AIM (Reach, Effectiveness, Adoption, Implementation, Maintenance) framework [35], we will also use quantitative data and qualitative data to assess the potential reach of MHMC to patients outside the trial setting, participant fidelity to the intervention, and factors that may affect widespread and sustained implementation of MHMC in real-world clinical practice.

### The Intervention

MHMC is a web-based app that can be viewed on mobile phones, laptops, desktop computers, or tablets. It is not a native app that requires downloading from iOS or Android. Informed by our preliminary data, an explanatory framework informed by the Health Belief Model and the principle of contraceptive autonomy [28], and the Ottawa Decision Support Framework [36], we developed MHMC with a professional software team (Alfa Jango). This process included iterative cognitive interviews with potential end users (people with at least 1 index health condition who want to explore their contraceptive options).

**Figure 2:**
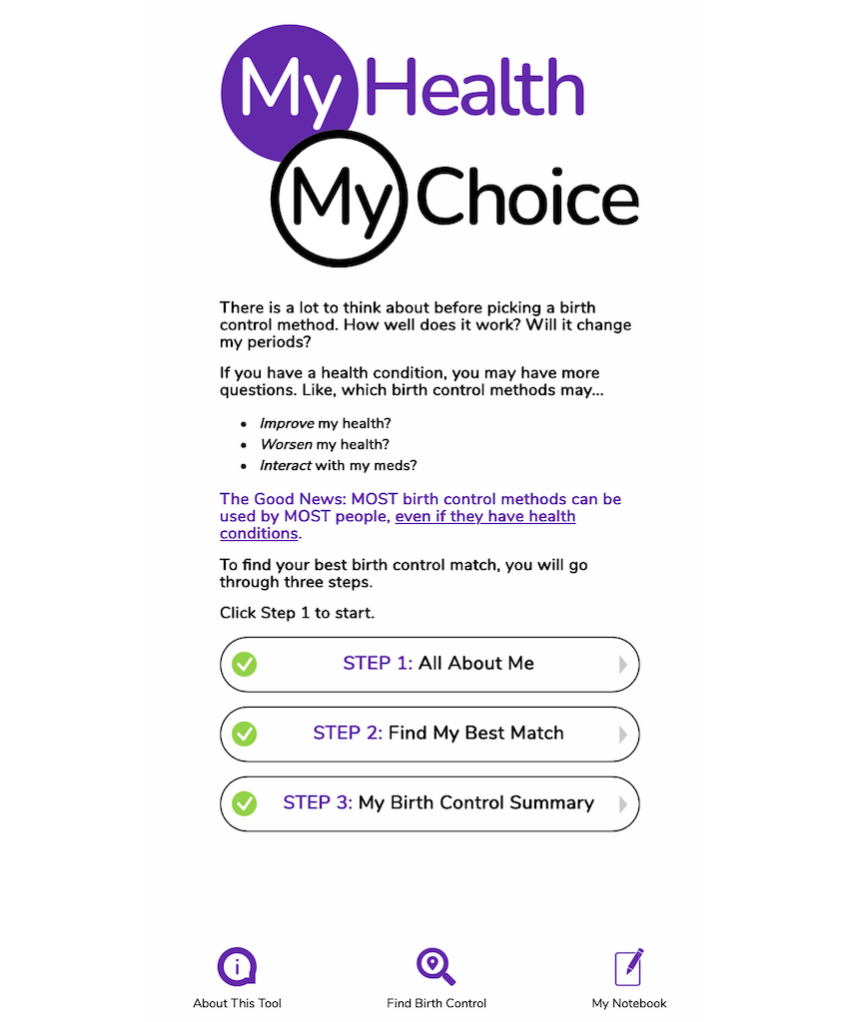
My Health, My Choice home screen for patients.

The patient version consists of 3 steps (Figure 2). While using the tool, patients can write down any questions or concerns in a “My Notebook” feature that saves their responses. In Step 1 (All About Me), patients manually indicate if they have any of 60 index health conditions and relevant characteristics, such as smoking status or postpartum status. Most of the index conditions are chronic in nature (eg, migraine headaches, kidney disease, and multiple sclerosis), but they also include acute conditions (eg, recent thromboembolic or cardiovascular event) and past pregnancy-related conditions (eg, gestational diabetes and preeclampsia) that may affect perceived risks and benefits of contraception and unplanned pregnancy. Patients also indicate if they are taking any of the 50 medications associated with fetal defects, interactions with hormonal contraception, or both (Multimedia Appendix 1). To prevent user fatigue and improve readability, similar medications are grouped (eg, statins and neuromodulators) and presented over several screenshots. Patients then receive tailored contraceptive education that uses 6th-8th grade English to explain theoretical or known risks and benefits of different methods in the context of their index health conditions and medications. In Step 2, patients are shown contraceptive methods grouped by a traffic-light color scheme and text to simply translate US MEC medical risk categories for layperson comprehension: (1) Category 1 and 2: “Safe. Use any of these” (green bar); (2) Category 3: “Caution. These methods may be risky for you. Use only if the benefits outweigh the risks to you. Talk to your clinician. (yellow bar).” (3) Category 4: “Stop. Do not use these.” (red bar). Patients can click on each method to learn about relevant features (eg, contraceptive effectiveness, impact on periods, and side effects), add a method to their potential “Favorites,” and click on methods to compare them side-by-side. In Step 3, the tool generates a “My Birth Control Summary” that displays the individual’s health conditions, medications, contraceptive favorites, and questions for their clinician from “My Notebook.” No personal identifiers are included in this screenshot (Multimedia Appendix 2).

**Figure 3:**
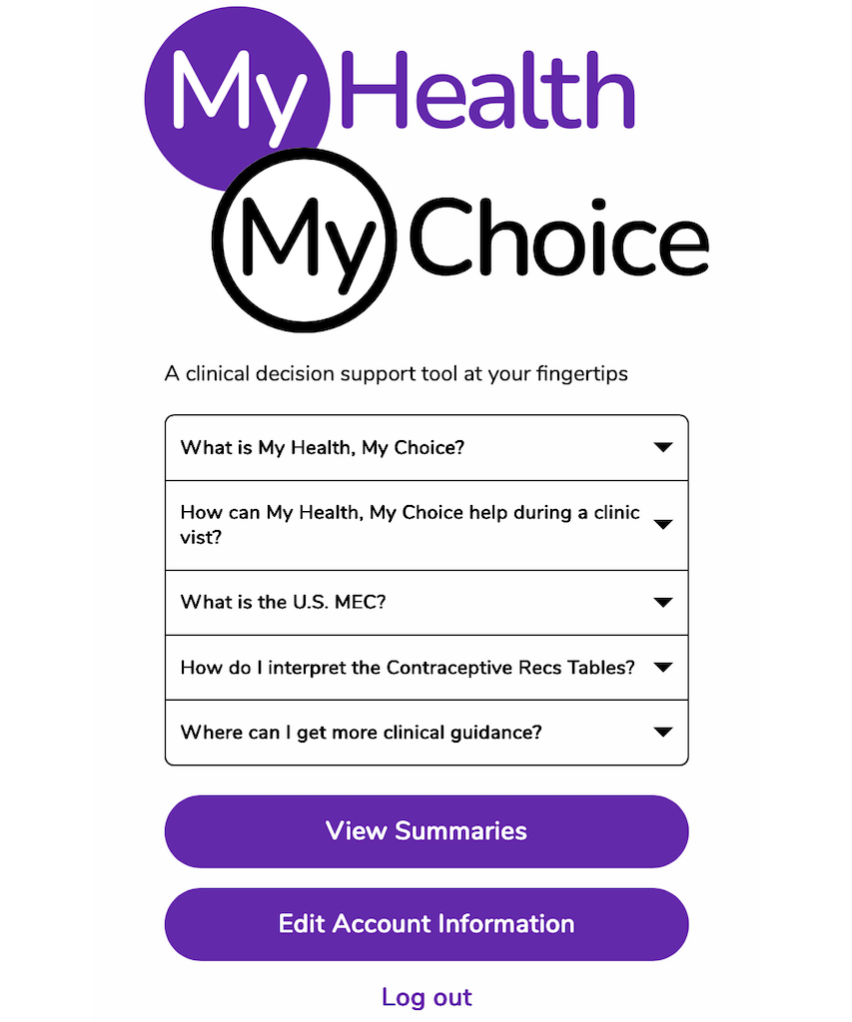
My Health, My Choice home screen for clinicians.

The clinician version (Figure 3) is designed to provide point-of-care support to help clinicians counsel individuals about the safety of different contraceptive methods in the context of their health conditions, medications, and personal concerns. Clinicians are prompted to review each enrolled patient’s “My Birth Control Summary” before or during the clinic visit. Clinicians are not required to enter any patient health information. This approach minimizes clinician burden and, more importantly, ensures that the discussion is driven by patient concerns and priorities. Finally, clinicians are provided with curated snapshots and brief text descriptions of sections of the US MEC Chart that highlight conditions and medications relevant to the index patient (Multimedia Appendix 3).

### Inclusion and Exclusion Criteria: Clinics, Clinicians, and Patients

Eligible clinics must (1) provide contraceptive counseling and services, including provision of or referral for intrauterine devices (IUDs), implants, and permanent contraception (tubal surgery or vas deferens surgery); (2) identify a clinician to serve as a clinic lead for study activities; (3) agree to be randomized; and 4) cede to oversight by the University of Michigan (UM) Institutional Review Board (IRB) per the National Institutes of Health (NIH) and federal single IRB mandate (US Code of Federal Regulations Part 46.114) [37]. Clinics are not eligible if they share providers or contraceptive quality improvement or educational initiatives with another clinic that is currently enrolled in the trial. Eligible clinics must operate as independent units to avoid the risk of contamination or bias across clinic sites [38].

Eligible clinicians must (1) be a licensed certified nurse midwife, nurse practitioner, physician assistant, or physician. Resident physicians who have completed 1 year of internship are eligible (interns are excluded) and (2) provide contraceptive services, including provision of or referral for IUDs, implants, and permanent contraception (tubal surgery and vasectomy).

Eligible patients must (1) be aged 18-49 years; (2) be assigned female sex at birth (regardless of gender identity); (3) be able to read in English; (4) have access to a mobile phone, computer, or tablet with internet capabilities; (5) be fertile (have an intact uterus and at least 1 ovary, be premenopausal, have never had permanent contraception surgery, and have no known medical reasons for current infertility, such as use of ovarian suppression medications); (6) have at least 1 index health condition, take an eligible medication, or both (Multimedia Appendix 1; (7) not be currently pregnant; and (8) have a desire to start, switch, or add a contraceptive method for the purpose of preventing pregnancy. Patient exclusion criteria include patients who expect to try getting pregnant in the next 12 months or are presumed infertile for any reason (eg, postmenopausal, hysterectomy, or chemotherapy). During our recruitment process, we explicitly clarify to clinics, clinicians, and patients that transgender patients and postpartum patients are eligible for the trial as long as they meet all other eligibility criteria noted above. Of note, we are developing a Spanish version of the MHMC tool and anticipate expanding eligibility to Spanish-speaking patients in 2026.

### Recruitment, Screening, and Enrollment: Clinics, Clinicians, and Patients

To successfully enroll clinics, the study team is partnering with the DARTNet Institute, a national practice-based research network with over 1300 individual primary care physicians and clinician members representing more than 850 clinical practices in all 50 states within the United States. Clinics will be recruited via email, referrals through professional networks, and recruitment activities at professional conferences. If a clinic expresses interest, the DARTNet Institute team will meet with clinic leaders to ensure the clinic meets eligibility criteria prior to enrollment. Each enrolled site will have its own clinic team made up of 2-4 individuals, led by a clinician, who will be responsible for study activities.

Potentially eligible clinicians will have several opportunities to learn about the study through videoconference meetings, study flyers, the study website, direct communications with the clinic team, and customized emails. Clinicians who are interested in participation will complete an electronic informed consent via SignNow (airSlate Inc), a digital platform in compliance with the Health Insurance Portability and Accountability Act (HIPAA) of 1996.

The clinic team will identify potentially eligible patients through electronic medical record (EMR) screening of upcoming patient visits (1-4 weeks in advance to allow time to contact and enroll patients with local adaptations per each site’s typical scheduling norms). Visits must be scheduled with a clinician who has enrolled in the study to allow for valid interpretations of the impact of an intervention that targets both patients and clinicians. Eligible visit types include preventive health, gynecologic, postpartum, and follow-up or problem-based visits for any issue, including contraception. Potentially eligible patients will receive a study invitation by email, EMR portal message, text message, or letter, as determined by the clinic team. Study flyers will be posted throughout enrolled clinics to allow potentially interested patients to contact the study team directly. Patients who express interest in the study will undergo eligibility screening over the phone with a study team Research Assistant (RA). If patients meet eligibility, the RA will proceed with informed consent using SignNow.

### Patient Study Activities and Quantitative Data Collection

Enrolled patients will complete an electronic baseline survey to report demographics, health history, past and current contraceptive use, contraceptive knowledge, and the secondary and exploratory outcomes (see “Quantitative Outcomes and Analysis” section). To mitigate the risk of differential dropout, recruitment bias, or bias in baseline assessments, we will inform participants of their clinic group assignment after their consent and baseline surveys have been completed [39]. Although this approach entails temporary withholding of information, it is consistent with the Ottawa Statement on the Ethical Design and Conduct of Cluster Randomized Trials [39].

Patients in both the intervention and control arms will receive a web link to a brief video that explains “What to Expect” regarding study activities. Only those in the intervention arm will receive web links to the MHMC tool, a brief video that reviews how to log in and navigate the tool’s features, and a posttool survey to report their experience using MHMC.

**Figure 4:**
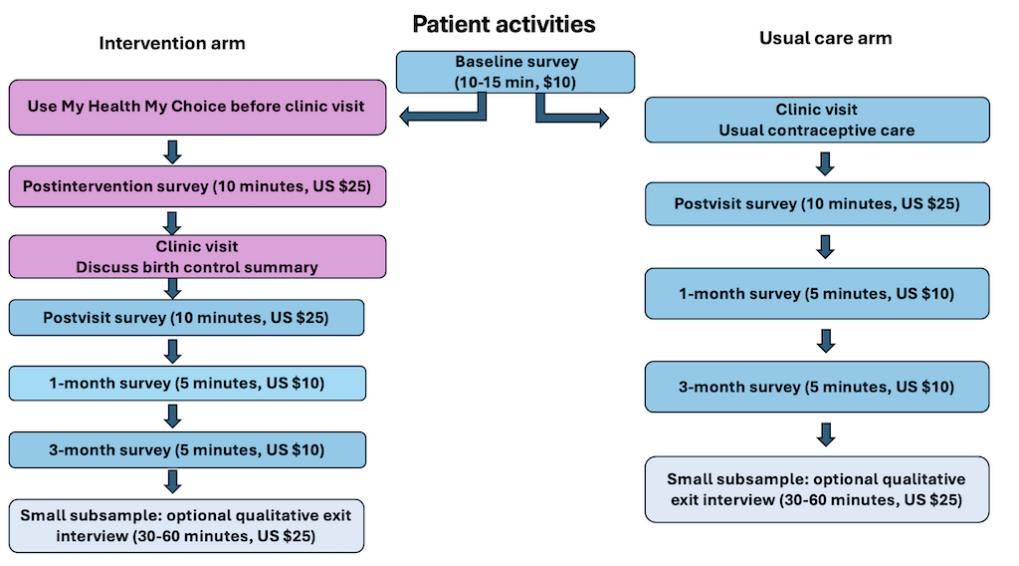
Patient Activities Flow Diagram.

The study team will encourage all patients to attend their upcoming clinic visit. Immediately after the clinic visit, patients will receive a postvisit survey and be asked to complete this within 5 days. They will be asked to complete surveys 1 month and 3 months after the clinic visit. Patient surveys require 5-15 minutes to complete. The study team will invite approximately 30 patients who complete study participation to do an optional 1-hour qualitative exit interview by phone or videoconference (refer to “Qualitative Data Collection and Sampling Considerations” section). Patients will receive up to US $100 in cash remuneration for completion of study activities (Figure 4).

### Clinician Study Activities and Quantitative Data Collection

**Figure 5:**
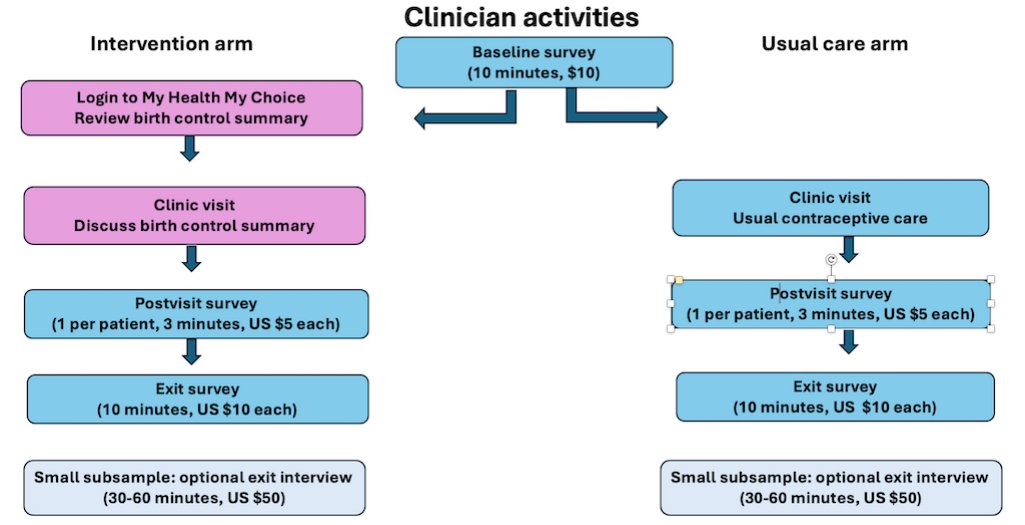
Clinician Activities Flow Diagram.

All enrolled clinicians will complete an electronic baseline survey and exit survey about their scope of contraceptive practice and knowledge of contraceptive care in the context of diabetes, hypertension, migraine headaches, and multiple risk factors for cardiovascular disease (common clinical scenarios). Clinicians in both arms will receive a web link to a video that reviews “What to Expect” regarding study activities. Only clinicians in the intervention arm will receive web links to the MHMC tool and a brief video that reviews how to navigate the tool’s features. After each clinic visit with an enrolled patient, clinicians will complete a 3-minute postvisit survey. The study team will invite approximately 30 clinicians at the end of the study to do an optional 1-hour qualitative exit interview by phone or videoconference (refer to “Qualitative Data Collection and Sampling Considerations” section). Clinicians will receive up to US $75 in cash remuneration for study activities (Figure 5).

### Clinic Study Activities and Quantitative Data Collection

After patients exit the study, the Clinic Team will conduct an EMR review of each enrolled patient’s chart to corroborate 3-month study outcomes, including contraceptive use (contraceptive prescriptions, IUD, or implant insertions) or plans to obtain IUDs, implants, or tubal surgery for themselves or vasectomy for their partners (scheduled visits and referrals).

### Qualitative Data Collection and Sampling Considerations

To achieve the qualitative and mixed methods part of Aim 2, we will purposefully sample patients [40] across US MEC Categories 1, 2, 3, and 4). We will oversample patients who report the use of Category 3 to understand factors that led to the use of a method that should be avoided except under special circumstances. As an example, if a patient with well-controlled hypertension reports using a combined hormonal pill (Category 3 use), does this reflect oversight or unawareness of the risks by the patient, the clinician, or both? Or did the patient and clinician carefully review the pros and cons in light of the patient’s other comorbidities, age, and tolerance for risk? To what extent, if at all, did use of the intervention contribute to this process? While US MEC Categories will primarily drive qualitative sampling, we will also seek perspectives across a range of chronic conditions (cardiovascular, endocrine, psychiatric, neurologic, and autoimmune) and contraceptive method types whenever feasible, and across both arms. We also aim to purposefully sample clinicians who provided care to the index patients who agree to be interviewed, which will allow for comparison of patient and clinician perspectives on the clinic discussion and decision-making process.

To prevent the risk of affecting participant behavior or survey responses, we will conduct patient interviews right after they exit the study at 3 months and clinician interviews at the end of their clinic’s trial participation. A trained qualitative RA will conduct 30- to 60-minute interviews with participants over the phone or via videoconference. All audio and video recordings will be uploaded immediately to a secure cloud server. A professional transcriber will transcribe exit interviews verbatim. Personal identifiers for people and places will be removed and replaced with pseudonyms.

Theoretical saturation refers to the point at which data analysis reveals no further significant findings relevant to the research aims and is commonly used in qualitative research to determine sample sizes [41]. Based on our exit interviews from our pilot study and best practices in qualitative sampling [41,42], we anticipate that interviewing 2 patients and 2 clinicians per clinic (about 60 interviews total, 30 patients and 30 clinicians) will provide high-quality data to achieve theoretical saturation. Upon reaching theoretical saturation, we will stop collecting interview data.

### Power and Quantitative Sample Size

The primary outcome is the proportion of participants reporting contraceptive nonuse at 3 months. Based on national estimates [10,11,43,44] and our screening protocol, we anticipate 30% (217/726) of participants at baseline will report contraceptive nonuse. A previous cluster RCT of a contraceptive decision tool in outpatient clinics found a clinic-level intracluster correlation (ICC) of 0.02 [16]. We conservatively estimate an ICC of 0.05. Prior studies have estimated ICCs generally <0.04 for patient-reported outcomes in maternal health and sexual behavior among reproductive-aged women [45-47]. To reduce our estimated ICC, we have taken several precautions. At the design level, we have defined clinic, patient, and clinician eligibility criteria to reduce heterogeneity and variance in estimates [48]. At the analysis level, we will conduct regression adjustment for key covariates [48]. To detect a difference of 20% in the proportion of contraceptive nonuse between the intervention arm and the control arm at 3 months, an effect size consistent with that of prior contraceptive trials [20,49,50], we will need to enroll 14 clinics (7 in each arm) and 45 participants in each clinic. Assuming an attrition rate of 15%, our enrollment goal is 726 patients (363 patients in each arm). We anticipate that we will enroll 5-10 clinicians per clinic, for a maximum total of about 140 clinicians. The allocation ratio will be 1:1 with attention to the balance of obstetrics and gynecology clinics in each arm, which will mitigate the impact of unequal distribution of sites that provide higher volume or scope of contraceptive services.

### Randomization Process, Allocation Concealment, and Blinding

To avoid potential contamination across providers and patients, we will randomize by clinic site.

Clinics will be randomized in even-numbered blocks (2,4) to ensure balanced allocation of group conditions. The lead biostatistician (AS) will generate the allocation sequence and assign clinics to the intervention arm or control arm. Due to the cluster-randomized design, allocation concealment is impractical, and it will not be possible to blind patients or clinicians. The study team will remain blinded to the group assignment when evaluating outcomes.

### Quantitative Outcomes and Analysis

The primary outcome is contraceptive nonuse at 3 months per patient self-report, consistent with prior contraceptive studies [10,51,52]. Individual contraceptive nonuse will be modeled as a binary outcome. We will use mixed-effects nested logistic regression to assess differences in 3-month outcomes between the study arms. The model will include an arm assignment categorical covariate and will be adjusted using patient-level covariates (baseline contraceptive nonuse, education, race, ethnicity, and parity) and clinician-level covariates (self-reported experience with contraceptive care and specialty type). Random intercepts for the clinic and for clinicians nested within the clinic will account for the clustering. Secondary analyses of contraceptive nonuse will include the same model with 1-month data and a subgroup analysis that only includes patients at risk for pregnancy, that is, those who report penile-vaginal sexual activity at 1 month and 3 months.

The secondary quantitative outcome is patient-reported contraceptive method use as classified by the US MEC. We will summarize US MEC Categories 1, 2, 3, or 4 among patients who report contraceptive use at 1 month and at 3 months with descriptive statistics. Chi-square tests will be calculated to compare frequencies between the intervention group and the usual care group.

The exploratory outcomes are hypothesized mediators of the intervention effect on the primary and secondary outcomes. First, contraceptive decisional conflict will be assessed with the DCS, a 16-item validated scale with 4 subscales [29] (0=no conflict; 100=greatest conflict). We slightly modified the wording of DCS items, when appropriate, to reflect contraceptive decisions rather than generic health decisions. Second, we will assess patient-reported quality of interaction with their contraceptive provider via the IQFP [30]. The IQFP contains 11 items that are assigned a score of 1-5 (1=poor, 2=fair, 3=good, 4=very good, and 5=excellent) by the patient to assess their clinician’s shared decision-making and interpersonal skills (total scores ranged from 11 to 55). We will conduct a mediation analysis as described by Baron and Kenny [53] with two models: (1) a logistic regression of the mediator (ie, DCS or IQFS, dichotomized) as a function of the primary outcome (contraceptive nonuse at 3 months), with a covariate for intervention versus control group, and (2) a logistic regression of the primary outcome (contraceptive nonuse at 3 months), adjusting for both the mediator (ie, DCS or IQFPS, dichotomized) and intervention versus usual care group.

We will conduct descriptive analyses on other patient, clinician, and clinic-level exploratory measures and statistical comparison tests as appropriate (eg, chi-square, Fisher exact, and *t* tests).

- Patient-level measures: We will categorize patient-reported contraceptive use by method type and method effectiveness (highly effective, moderately effective, and less effective) to enable comparison with other studies. Patients will be asked to assess 6 knowledge questions regarding contraceptive safety (true, false, or I don’t know) at baseline, postdecision tool use (for intervention arm only), and postclinic visit. Patients will be instructed to report any pregnancies that occur during the study and at 1 month and 3 months. For any pregnancies reported, patients will be asked to describe their pregnancy intentions at the time of pregnancy using a standardized single item [54]. Patients who are still pregnant at the time of reporting will exit the study and seek follow-up care; they may contribute data up until the point of study exit.
- Clinician-level measures: We will assess clinician knowledge of the US MEC Guidelines at baseline and exit surveys with 5-point Likert-scale items regarding 4 clinical scenarios.
- Clinic-level outcomes: RE-AIM is an evaluation framework to assess an intervention’s potential to be translated to real-world settings [35]. We will assess each dimension of RE-AIM with quantitative data, qualitative data, or both:
- Reach: We will collect demographics of participants offered the intervention and those who used the intervention, and these will be compared with those of the usual contraceptive care arm, as well as the general US population at risk for unplanned pregnancy (quantitative).
- Efficacy: We will examine the primary outcome of contraceptive nonuse (quantitative) in the context of patient and clinician perspectives on the intervention’s impact (qualitative).
- Adoption: We will report the percentage of patients and clinicians in the intervention arm who used the decision tool intervention.
- Implementation: We will report patient and clinician fidelity to the intervention and what natural adaptations were made (quantitative and qualitative).
- Maintenance: We will summarize patient- and clinician-reported facilitators, barriers, and suggested changes to sustain use of the intervention in clinical practice beyond the clinical trial (qualitative).

### Qualitative Analysis

Using qualitative and mixed methods analysis software (MAXQDA, ERBI GmbH) [55], the Principal Investigator (PI;JPW) and a trained qualitative RA will conduct thematic analysis as described by Braun and Clark [56]. Analysis of patient interviews and clinician interviews will be conducted separately. First, we will independently read the first several transcripts and assign codes to text segments. We will iteratively repeat this process with subsequent transcripts and merge and revise codes as needed. The final codes and code definitions will be organized in a consensus codebook. In the second phase of qualitative analysis, the PI and RA will merge codes to advance from descriptive codes to analytic codes and themes. Thematic findings within and across patients, clinicians, and clinics will be compared. Upon reaching theoretical saturation, such that no significant new insights are gained with respect to Aim 2, qualitative data collection will stop. TG will assist as a third analyst to resolve differences in interpretations and help decide when qualitative data collection can stop. We will triangulate thematic findings from the patient interviews and the clinician interviews to explore confirming and disconfirming findings.

### Mixed Methods Analysis

**Figure 6:**
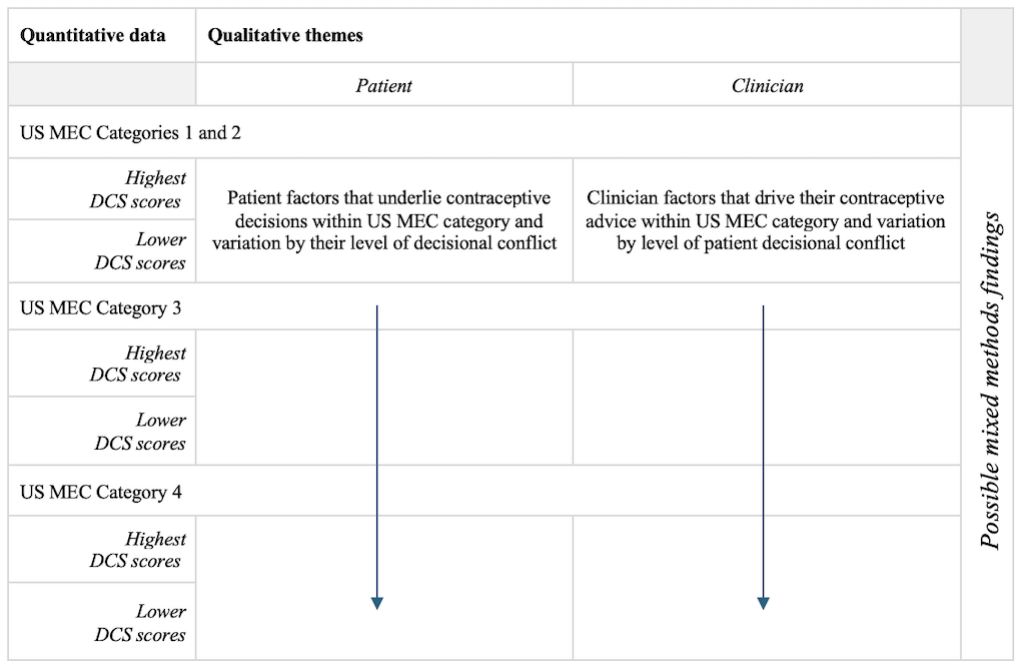
Mock-up example of joint display.

Mixed methods in health research involve the integration of quantitative and qualitative data to gain a greater understanding of the phenomenon than can be obtained with either type of data alone [34]. We will integrate the quantitative data findings about US MEC Categories with the qualitative themes to better understand how and why the intervention and other contextual factors may have affected decision-making across US MEC Categories, again, with a focus on Category 3 cases. To do so, we will create a joint display, a mixed methods technique to array quantitative and qualitative findings side-by-side and draw out inferences and generate hypotheses (Figure 6) [34].

### Ethical Considerations

The UM IRB approved the study protocol (HUM00234187). This trial will be conducted in compliance with this protocol, the International Council on Harmonisation Good Clinical Practice, and applicable state, local, and US Code of Federal Regulations (45 CFR Part 46, 21 CFR Part 50, 21 CFR Part 56, 21 CFR Part 312, and/or 21 CFR Part 812), and the National Institute of Child Health and Human Development (NICHD) Terms and Conditions of Award. All personnel involved in the conduct of this study have completed human participants protection and good clinical practices training. All study data will be collected and managed using REDCap (Research Electronic Data Capture; Vanderbilt University) surveys hosted at the UM. REDCap is a secure, web-based software platform designed to support data capture for research studies [57]. Clinician and patient participants receive cash compensation for completion of each study activity (see Figures 4 and 5).

## Results

This study was funded in September 2023. We anticipate data collection to start in October 2025 and expect to conclude data collection in October 2028. A Data Safety Monitoring Board of 4 independent experts with combined expertise in biostatistics, maternal and child health, and family planning will assess the trial’s progress and monitor participant safety. Our findings will be published in peer-reviewed journals. We anticipate expanding eligibility to Spanish-speaking patients in 2026 after creating a linguistically appropriate Spanish version of the decision tool.

## Discussion

### Principal Findings

This study aims to evaluate whether a web-based decision tool will decrease contraceptive nonuse by improving patient-reported decisional conflict and the quality of interactions with their contraceptive providers. To our knowledge, this will be the first RCT to focus on the contraception decision support needs of people with health conditions—a group at higher risk of adverse events (albeit low absolute event rates) from hormonal contraception use and pregnancy-related complications than those without health conditions [11,12]. We expect this study to advance our understanding of the impact of contraceptive decision support tools on patients’ perceptions of their clinicians’ ability to provide comprehensive information, listen to their concerns, respect patient autonomy, and engage in shared decision-making [30]. This study will also generate new knowledge regarding how patients and clinicians deliberate risk-benefit trade-offs of contraceptive use in light of ongoing health considerations.

### Study Strengths

A key strength of this study is its cluster RCT design, which is a rigorous approach to mitigate contamination, that is, inadvertent exposure to the intervention among participants in the control arm [39]. Contamination across patients who seek care at the same clinic is possible, though less likely. The greatest risk of contamination from exposure to MHMC is among clinicians who work together at the same clinic; randomization at the clinic level will mitigate this risk. A second strength of the study design is the use of qualitative and mixed methods to disentangle the complexities of contraceptive decisions in the context of health considerations that cannot be ascertained via quantitative survey items alone. We anticipate this approach will be most useful for probing Category 3 situations when the use of a method should be avoided unless there is no other appropriate or preferred method available. Finally, this trial will be conducted in real-world clinic settings in diverse geographic regions of the United States, including nonacademic sites, which will improve the generalizability of our findings.

### Limitations

The limitations of this study include the heterogeneity of eligible health conditions, which may dilute the overall effect size for the primary outcome (contraceptive nonuse). However, the posited behavioral mechanisms that support this intervention (decreased decisional conflict and improved interactions with clinicians) should, in theory, operate across different health conditions. We acknowledge that including clinicians with a diverse range of expertise in chronic disease management, family planning, and obstetrics and gynecology may introduce performance bias. We will conduct subanalyses to explore this possibility. Finally, a major trial limitation is the exclusion of non–English-speaking patients. Development and piloting of a Spanish version of MHMC is underway, and we anticipate recruiting Spanish-speaking patients in 2027.

### Conclusions

In summary, this mixed methods cluster RCT will inform clinical care and best practices to support people’s reproductive priorities, reduce contraceptive nonuse, and improve reproductive health outcomes. We will share our study findings with participating clinics and their patients through our study website, newsletters, and national conferences, and with the scientific community through peer-reviewed journals and scientific presentations. This protocol will improve the rigor and reproducibility of future trials of contraceptive decision support tools.

## Appendix

Multimedia Appendix 1 My Health My Choice: List of index health conditions, characteristics, and medications.

Multimedia Appendix 2 My Health My Choice: My birth control summary example.

Multimedia Appendix 3 My Health My Choice: Clinician point of care US Medical Eligibility Criteria for Contraceptive Use example.

Multimedia Appendix 4 Peer review report from the CMGC - Clinical Management in General Care Settings Study Section, Healthcare Delivery and Methodologies Integrated Review Group (National Institutes of Health, USA).

## Abbreviations

CDC: Centers for Disease Control and Prevention
CHC: combined hormonal contraceptives
DCS: Decisional Conflict Scale
EMR: electronic medical record
HIPAA: Health Insurance Portability and Accountability Act of 1996
ICC: intracluster correlation
IRB: Institutional Review Board
IQFP: Interpersonal Quality of Family Planning Scale
IUD: intrauterine device
MHMC: My Health, My Choice
NICHD: National Institute of Child Health and Human Development
NIH: National Institutes of Health
PI: Principal Investigator
RA: Research Assistant
RCT: randomized controlled trial
RE-AIM: Reach, Effectiveness, Adoption, Implementation, Maintenance
REDCap: Research Electronic Data Capture
SPIRIT: Standard Protocol Items: Recommendations for Intervention Trials
UM: University of Michigan
US MEC: United States Medical Eligibility Criteria for Contraceptive Use

## Data Availability

The datasets generated or analyzed during this study will be made available beginning 1 year after publication of trial findings and for an additional 36 months. After this 36-month time period, the quantitative data will be available in the UM’s Deep Blue Data Repository, and the qualitative data will be available in the Qualitative Data Repository, but without investigator support other than deposited metadata.
