# Supplementary figures and images for: A Web-Based Contraception Decision Tool for Individuals With Health Conditions in US Outpatient Clinics: Protocol for a Mixed Methods Cluster Randomized Controlled Trial
Source: JMIR Res Protoc. 2025 Dec 29;14:e71101. doi: 10.2196/71101 (PMC12796879; doi:10.2196/71101)

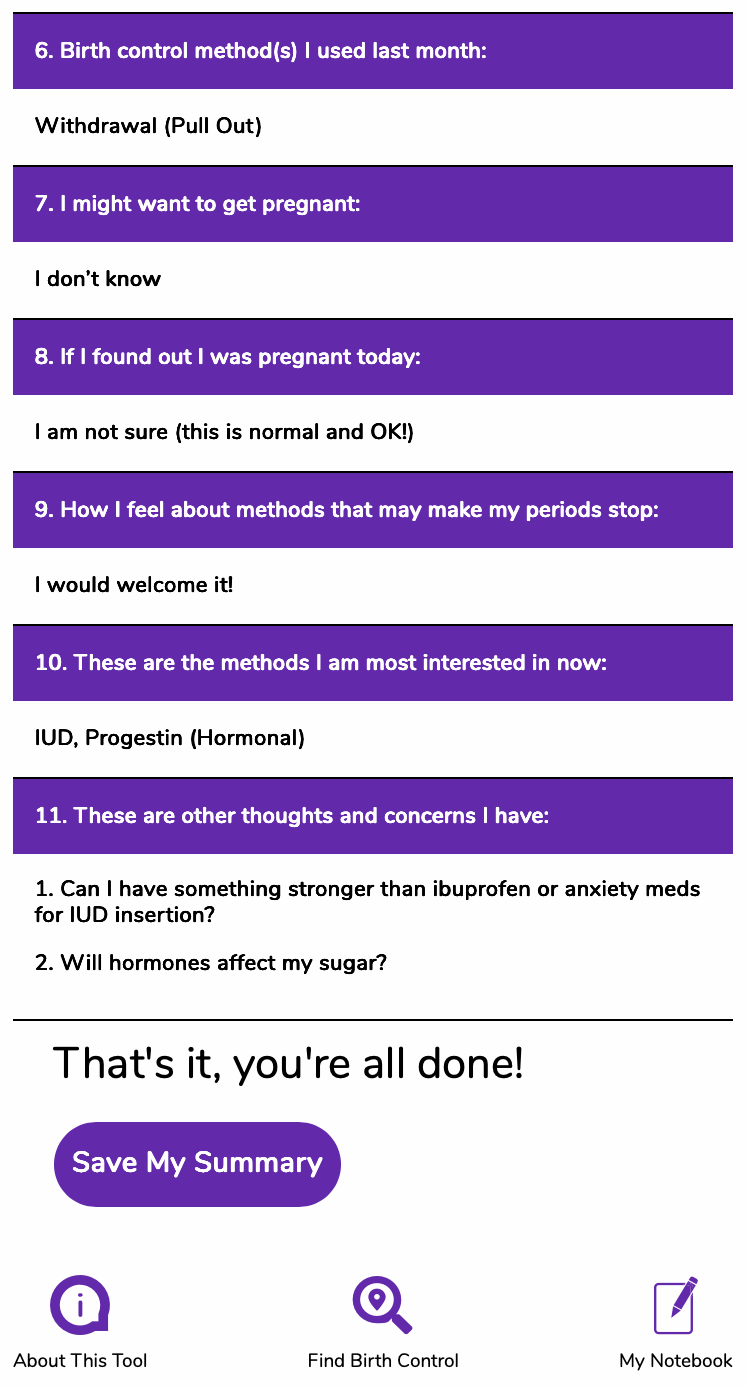


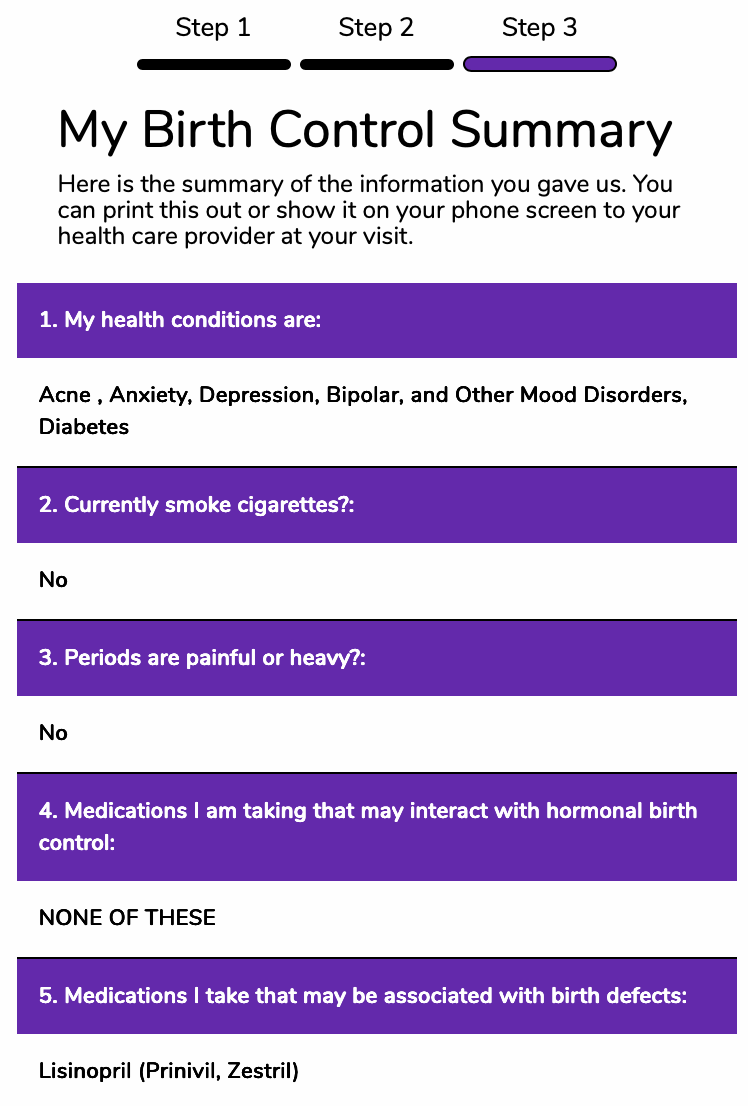

Supplement: Multimedia Appendix 2 [file resprot_v14i1e71101_app2.docx]

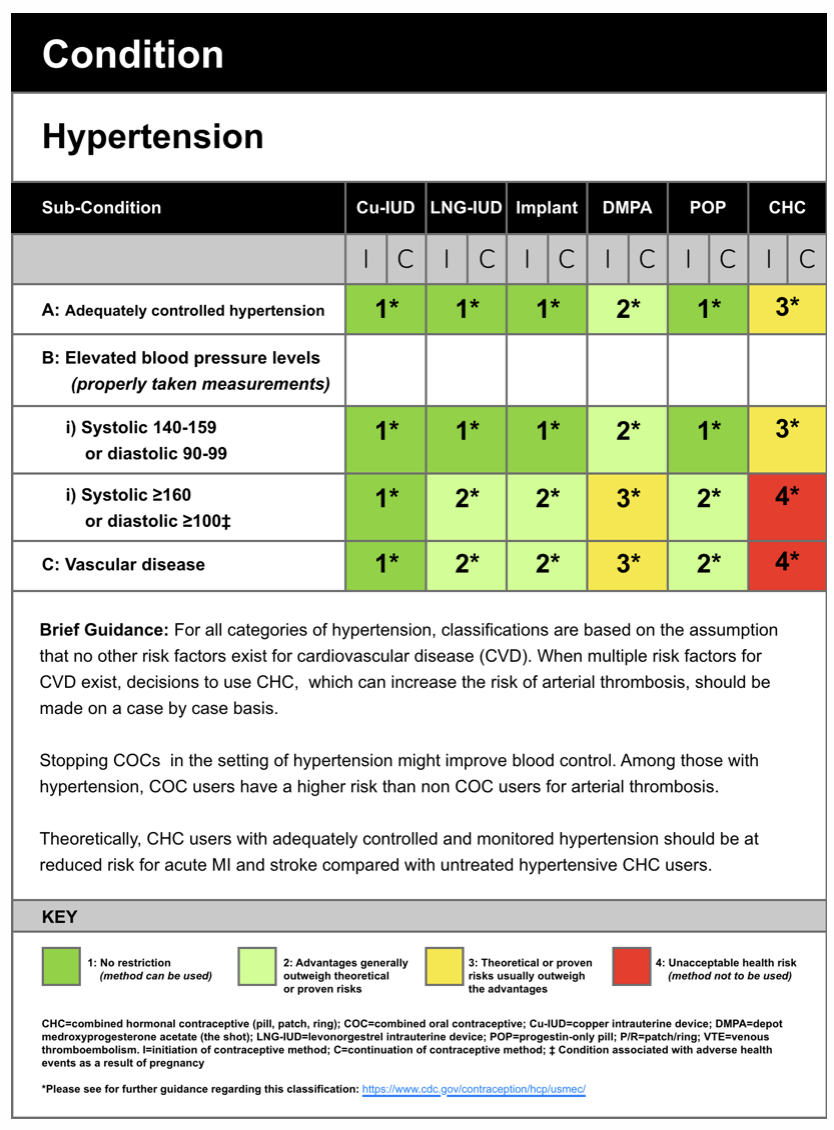

Supplement: Multimedia Appendix 3 [file resprot_v14i1e71101_app3.docx]
