# Supplementary material for: A Web-Based Contraception Decision Tool for Individuals With Health Conditions in US Outpatient Clinics: Protocol for a Mixed Methods Cluster Randomized Controlled Trial
Source: JMIR Res Protoc. 2025 Dec 29;14:e71101. doi: 10.2196/71101 (PMC12796879; doi:10.2196/71101)
Supplement: Multimedia Appendix 4 [file resprot_v14i1e71101_app4.pdf]

SUMMARY STATEMENT
(Privileged Communication)

PROGRAM CONTACT:
Dr Ronna Popkin
301-827-5121
ronna.popkin@nih.gov

Release Date: 06/24/2022
Revised Date:

Application Number: 1 R01 HD110570-01

Principal Investigator

WU, JUSTINE P

Applicant Organization: UNIVERSITY OF MICHIGAN AT ANN ARBOR

Review Group: CMGC
Clinical Management in General Care Settings Study Section

Meeting Date: 06/13/2022
Council: OCT 2022
Requested Start: 09/01/2022
RFA/PA: PA20-183
PCC: PDB -RP
Dual IC(s): NR

Project Title: Improving contraceptive decision support for individuals with chronic conditions

SRG Action: Impact Score:33 Percentile:17
Next Steps: Visit https://grants.nih.gov/grants/next_steps.htm
Human Subjects: 30-Human subjects involved - Certified, no SRG concerns
Animal Subjects: 10-No live vertebrate animals involved for competing appl.
Gender: 1A-Both genders, scientifically acceptable
Minority: 1U-Minorities and non-minorities, scientifically unacceptable
Age: 3A-No children included, scientifically acceptable

Project Year	Direct Costs Requested	Estimated Total Cost
1	574,967	894,229
2	574,962	894,221
3	574,965	894,225
4	574,997	894,275
5	574,977	894,244
TOTAL	2,874,868	4,471,194

ADMINISTRATIVE BUDGET NOTE: The budget shown is the requested budget and has not been adjusted to reflect any recommendations made by reviewers. If an award is planned, the costs will be calculated by Institute grants management staff based on the recommendations outlined below in the COMMITTEE BUDGET RECOMMENDATIONS section.
NEW INVESTIGATOR

1R01HD110570-01 WU, JUSTINE

COMMITTEE BUDGET RECOMMENDATIONS
INCLUSION OF MINORITIES PLAN UNACCEPTABLE
NEW INVESTIGATOR

RESUME AND SUMMARY OF DISCUSSION: This application proposes to evaluate the efficacy of a mobile health intervention to improve contraceptive decision making among individuals with pre-existing chronic conditions. Developing an intervention to promote quality contraception decision making among individuals with multiple chronic conditions meets a significant need as decision making is more medically and psychosocially complex for this patient population. This project is led by a strong research team with a history of prior collaboration. However, the discussion of the significance could be strengthened with a description of how the Ottawa Decision Support Framework will guide the development of the mobile health tool, and additionally, the team does not include any expertise in decision making. The focus on contraception for individuals with chronic conditions is innovative. Strengths in the approach include the use of a mixed methods research strategy, such as the provider assessments of Medical Eligibility Criteria adherence, patient and clinician exit interviews, and recorded patient-clinician interactions. Reviewers also noted weaknesses in the approach, including the sampling strategies for the clinician or patient subgroups for exit interviews is not well described and vague. Overall, this is an innovative application that will have a high impact on the development of a mobile health tool to support contraceptive decision making among patients with multiple chronic conditions.

DESCRIPTION (provided by applicant): Individuals with pre-existing chronic conditions are more likely to experience unplanned pregnancy and pregnancy-related complications than their peers without chronic conditions. Because contraceptive nonuse (not using any contraceptive method) poses the greatest risk of unplanned pregnancy and is more prevalent among those with chronic conditions, there is an urgent need for contraceptive interven- tions for this group. Contraceptive decision-making is a critical antecedent to contraceptive use, and a highly personal process. A significant care gap is the lack of tools that address the unique contraceptive decisional needs of patients with chronic conditions. Patients should know which methods may posi- tively or negatively affect their chronic disease symptoms (e.g., anemia) or self-management (e.g., weight change). Patients should also be informed if their chronic condition poses a health contraindica- tion to a specific method, and if so, which other methods they can use safely. To make informed contra- ceptive decisions, patients desire clinician advice that is evidence-based and responsive to their needs. However, clinicians do not routinely adhere to contraceptive guidelines or elicit patient preferences. To address these gaps, we developed a user-tested mobile tool, My Health, My Choice (MHMC) to simplify contraceptive decisions for patients with chronic conditions and to promote clinician adherence to con- traceptive guidelines in a patient-centered manner. MHMC is innovative because it will be the first con- traceptive decision support tool that targets both patients with chronic conditions and their clinicians. Our long-term goal is to improve reproductive health through patient-centered contraceptive interven- tions in general care settings. To build upon our promising preliminary findings, the study objective is to evaluate the efficacy of MHMC in a two-arm, parallel cluster-randomized trial using mixed methods to compare MHMC (intervention) to usual contraceptive care (control) in 14 primary care clinics that pro- vide contraception. Our hypothesis is that MHMC will improve patient-reported quality of contraceptive decision-making and experience of contraceptive care from their clinicians, which will lead to increased use of contraception and a decrease in nonuse. The study aims are: Aim 1: (Primary outcome) To eval- uate the impact of MHMC on contraceptive nonuse. Aim 2: (Secondary outcome) To assess the impact of MHMC on adherence to contraceptive guidelines using mixed methods. Aim 3: (Exploratory): To ex- plore potential mediators of the effects of MHMC on contraceptive nonuse. The expected outcomes are new findings regarding the efficacy of a novel contraceptive decision tool for individuals with chronic conditions in primary care.

The research will inform the development of scalable, patient-centered con- traceptive interventions for medically at-risk patients in real-world clinical practice.

PUBLIC HEALTH RELEVANCE: Individuals with chronic conditions are more likely to not use contraception and experience un- planned pregnancy yet lack resources that address their unique contraceptive decisional needs. To address this pressing public health and clinical issue, this project will evaluate a mobile contraceptive decision support tool that helps patients choose contraception in context of their chronic conditions and personal priorities. The project's focus on contraception for a medically at-risk population is aligned with NICHD's Strategic Plan to support people's reproductive priorities, reduce contraceptive non-use, and improve maternal health and reproductive health outcomes. .

CRITIQUE 1

Significance: 3
Investigator(s): 1
Innovation: 2
Approach: 4
Environment: 1

Overall Impact: This new R01 proposal aims to evaluate the efficacy of a web-based, mobile-optimized decision tool, called *My Health My Choice,* on contraceptive nonuse for individuals with chronic conditions who are receiving primary care. Contraceptive nonuse represents an area of reproductive health unmet need among individuals with chronic conditions, who subsequently experience greater risk of unplanned pregnancy and pregnancy morbidity. In addition, the needs of individuals with chronic conditions require more complex contraception decision making. Thus, the investigators have developed this tool in order to simplify decisions for both patients and clinicians. The study will use a 2-arm, cluster RCT approach in which Aim 1 is to evaluate the MHMC intervention's effect on contraceptive nonuse and Aim 2 is to evaluate the impact of MHMC on adherence to contraceptive guidelines (via patient-reported outcomes, exit interviews, and recordings of patient-clinician interactions). An exploratory Aim 3 is to evaluate potential mediators of the effects of MHMC on contraceptive nonuse. The trial will take place at 14 diverse clinical practices among adult women who do not desire pregnancy. The investigators' long-term goal is to inform the development of a patient-centered intervention for individuals with chronic conditions that can ultimately be used in real-world practice. Significance of this proposal is high, given the importance of contraception for all individuals but particularly those with chronic conditions, the potential impact of an efficacious decision support tool, and the rationale for use of a contraception support tool in this population. Rigor of prior research is high. There are minor-to-moderate limitations of significance, such as concerns regarding the heterogeneity of the chronic conditions, the limitation of focusing on patients who are already engaged in the healthcare system, and the lack of attention of MHMC to partner involvement. The investigators are highly prepared for this proposal and the team has all the necessary expertise. Innovation is also high for a number of reasons. Additionally, the approach has many strengths. The use of a cluster RCT conducted in diverse settings is a strength, as is the rigorous design of MHMC and the preliminary data. The proposal includes strong analysis plans and justification for features of the study design. The study has several features in place to support long-term dissemination and implementation plans, such as an implementation science-guided analysis of findings. There are moderate weaknesses, including unclear goals of Aim 3, lack of inclusion of prior adverse pregnancy outcomes as chronic conditions (given their known associations with long-term cardiometabolic health), and potentially excessive qualitative sampling. In summary, this very well-written, impactful, and

rigorous proposal is felt to have significant potential to achieve its stated goals. The team is well prepared, rigor of prior data is strong, and approach is solid. Thus overall impact is felt to be high.

1. Significance:

Strengths

- Contraception non-use among individuals with chronic conditions represents an area of unmet reproductive health need. The proportion of reproductive aged individuals who have chronic conditions is rising.
- Contraceptive decision making for individuals with chronic conditions is more medically and psychosocially complex for patients, and more complex for clinicians as well. Clinician adherence to guidelines is imperfect and patient preferences are not always elicited. Thus, there is a strong rationale for a decision aid for this population.
- Studying the uptake of contraception, along with improving pregnancy outcomes, are aligned with the NICHD priorities.
- Adherence to the CDC Medical Eligibility Criteria is suboptimal, and primary care physicians are an ideal health care group for an intervention regarding the MEC, since they already provide care for individuals with chronic conditions.
- Decision tools are evidence-based strategies to understand health options, and prior data on tools for contraception have suggested potential effects on contraceptive decision making (though mostly were among individuals without chronic conditions). The limitations of prior trials were identified by the investigators.
- The MHMC has a strong theoretical foundation.
- Prior work by the PI has informed the chronic condition-related concerns that women have about contraception and informed the MHMC development.
- Preliminary data from the PI's one-arm pilot study of 46 individuals suggested the MHMC intervention is acceptable and feasible in primary care settings.

Weaknesses

- The chronic conditions under investigation are highly heterogeneous, limiting the ability to determine if MHMC is more efficacious for some types of conditions than others.
- The nature of this proposal and the MHMC platform (to be used prior to appointments) means it will capture an already-engaged population (i.e., those who are already seeing primary care clinicians), leaving a missed opportunity for meeting the needs of those who may only be accessing subspecialty care, emergency care, or no care at all.
- Given the nature of family medicine, some individuals accessing primary care may actually be newly postpartum, but it is not clear if MHMC includes any tailoring for this population.
- MHMC does not appear to include any element of partner decision making support, which could be important for some individuals.
- Limited availability of MHMC to English-speaking users is a moderate weakness.

2. Investigator(s):

Strengths

- PI Dr. Wu is a family medicine physician with fellowship training in family planning who is currently on an NICHD K23, which she has used to obtain advanced training in clinical trials, mixed methods, and implementation science. She developed the *My Health, My Choice* tool, the decision aid that is to be investigated, during her K23 award. She has appropriate clinical and research experience to lead this proposal, including a successful funding and publication record in this area.
- Co-I Dr. Aikens is Dr. Wu's mentor on her K23 and an experienced health psychologist who has particular experience in behavioral interventions for individuals with chronic conditions. His mentorship will support Dr. Wu's transition to her independent investigator role.
- The team additionally includes experts in mobile health and health communication technologies (Buis), obstetrics and gynecology/complex family planning and contraceptive research (Dalton), qualitative and mixed methods research methodology (Guetterman and others), and biostatistics (Sen).
- A collaborator from the AAFP National Research Network will support the research implementation component of the study.
- The team has adequate prior collaboration expertise.

Weaknesses

- None identified

3. Innovation:

Strengths

- A decision aid that addresses both patients and their clinicians is particularly innovative.
- The focus on contraception for individuals with chronic conditions is innovative.
- The MHMC tool itself is innovative in several ways, including its interactive nature, being a mobile optimized, web-based tool (rather than requiring download to a specific platform), and the point-of-care clinician support that is available.

Weaknesses

- The study of contraception uptake is not necessarily novel (as noted by the investigators, at least 6 other RCTs have investigated contraception decision support tools).

4. Approach:

Strengths

- MHMC is an interactive mobile-optimized web-based decision support tool that does not require entering PHI (thus enhancing privacy) which patients can use prior to clinical interactions. The patient summary and clinician support components can then be viewed by the primary care provider.
- MHMC has gone through appropriate preparatory phases, including qualitative work to support the tool development, cognitive think alouds, and pilot testing. Pilot testing supports the readiness for expanded study.
- The team will partner with the AAFP NRN to recruit 14 clinics for this cluster RCT, which is a rigorous and appropriate study design. Other alternative study designs were considered and

rationale for their non-use was described. Overall the justification for and details about this study design were excellent.

- Clinic, clinician, and patient eligibility are described in detail with largely appropriate rationale.
- Power and sample size calculations are appropriate, as are the quantitative analysis plans for Aim 1.
- Aim 2 will use a combination of methods – MD assessment of MEC adherence, patient and clinician exit interviews, and recorded patient-clinician interactions – thus representing comprehensive, triangulated sources of data. Plans to rate the adherence are clear. Qualitative and mixed methods analysis plan is strong.
- Aim 3 will employ validated patient-reported outcomes – Decisional Conflict Scale and the Interpersonal Quality of Family Planning Scale.
- Implementation science framework (RE-AIM) will be used to evaluate MHMC. RE-AIM dimensions are described in detail.

Weaknesses

- Since recruited patients will have appointments in the upcoming 2-3 weeks, it seems that the amount of time between baseline, post-intervention, and post-clinic visit could be variable. It is also not clear if recruited patients must have an appointment specifically for contraception, or if their appointment can be any reason (while also having a need for contraception).
- Sampling strategy (regarding clinician or patient subgroups) for exit interviews is vague. Additionally the sample size estimates for the interviews seem potentially excessive.
- Chronic conditions under investigation include those recognize by the MEC and additional conditions associated with adverse pregnancy outcomes. Although heterogeneous (see above), the focus on the MEC is appropriate. However, one area of weakness is that prior adverse pregnancy outcomes (eg prior hypertensive disorders) are not considered among the chronic conditions.
- The goal of Aim 3 is to evaluate mediators of the effect of the intervention, though the rationale for choosing the DCS and IQFP (as opposed to any other measure) is not stated. It is also not clear if this aim will be useful if the intervention has no effect.
- Relatively little detail is provided about the clinician surveys.
- Minor: Feasibility and acceptability of the audio-recorded cancelling sessions is reported to be high though data are not provided. More detail on the analysis plans for these recordings was also needed (e.g., how the discussions will be coded and what topics are of interest).

5. Environment:

Strengths

- The University of Michigan and Michigan Medicine are excellent environments for this proposal. The Department of Family Medicine has a number of appropriate resources for the investigators and has supported the PI as an early career faculty member (including guaranteed research time after the completion of her K23 award). The Department of Obstetrics and Gynecology and several other Institutes also support this proposal and have many excellent resources.
- The American Academy of Family Physicians National Research Network also is an appropriate environment.

- Letters of support from the clinical sites are provided.

Weaknesses

- None identified

Study Timeline:

Strengths

- Appropriate. Includes phased clinic entry.

Weaknesses

- None noted by reviewer.

Protections for Human Subjects:

Acceptable Risks and/or Adequate Protections

- Well done protections in place for all parties.

Data and Safety Monitoring Plan (Applicable for Clinical Trials Only):

Acceptable

- Includes a DSMB

Inclusion Plans:

- Sex/Gender: Distribution justified scientifically
- Race/Ethnicity: Distribution justified scientifically
- For NIH-Defined Phase III trials, Plans for valid design and analysis: Scientifically acceptable
- Inclusion/Exclusion Based on Age: Distribution justified scientifically
- Although the proposal would have been strengthened by including minors, the investigators note that the MHMC tool was not developed for adolescents, and that their unique needs mean it may not be appropriate to include them.

Vertebrate Animals:

Not Applicable (No Vertebrate Animals)

Biohazards:

Not Applicable (No Biohazards)

Resource Sharing Plans:

Acceptable

Budget and Period of Support:

Recommend as Requested

Recommended budget modifications or possible overlap identified:

- Large budget with many personnel, but this may be appropriate for the scale of the proposal.

CRITIQUE 2

Significance: 4
Investigator(s): 3
Innovation: 1
Approach: 3
Environment: 1

Overall Impact: The objective of the proposed study is to evaluate a theory-informed and user-tested mobile contraceptive decision support tool for individuals with chronic conditions. This proposal addresses an important issue in women's health – avoidance of unplanned pregnancy. The innovation is high and the investigative team is strong. The approach has some very strong aspects such as the inclusion of 14 clinics, the large sample size, and the exploratory aim addressing potential mediators of the decision to use or not use contraception. The major weakness was related to the concept of decision making. There is no discussion of decision making theory in the significance and no one on the investigative team brings expertise in decision making.

1. Significance:

Strengths

- Health Belief Model and pilot study used to guide a focus on clinician counseling
- Strong pilot data demonstrating review by the CAC team responsible for disseminating the guidelines, 15 women who were interviewed, and data from a one-arm pilot study.
- Pilot data supports use of the Decisional Conflict Score as a mediator.

Weaknesses

- Ottawa Decision Support Framework is said to guide the MHMC tool, but the framework is not described and the manner in which the framework guided development of the tool is not described.
- Synchronized engagement of patients and clinicians during the clinic encounter is said to be the "theoretical basis" of the intervention but synchronized engagement is not discussed.

2. Investigator(s):

Strengths

- The PI designed the *My Health My Choice* decision tool during her K23. Since that early training period, she has had an impressive number of grants awarded. She has numerous awards and many excellent publications. She has already made notable scientific contributions.
- The PI is supported by a strong research team with expertise in primary care medicine, contraception, behavioral clinical trials, complex family planning and reproductive health, qualitative and mixed methods, mobile Health, chronic disease, and biostatistics.

Weaknesses

- No one with expertise in decision making is included on the team.

3. Innovation:

Strengths

- Focus on contraception, which has received relatively less attention as a strategy to improve maternal, infant, and reproductive health.
- Intervention simultaneously targets patients and clinicians.
- Intervention supports busy PCPs with information that is both patient-specific and harmonized with CDC Contraceptive Guidelines.
- Focus on women with chronic conditions.

Weaknesses

- None noted by reviewer.

4. Approach:

Strengths

- Study will be conducted in 14 geographically diverse U.S. primary care clinics.
- Randomizing at the clinic level rather than individuals to avoid contamination.
- Enrolling only women who *want* to discuss contraception.
- Two important potential mediators will be assessed: decision conflict and quality of care.
- After the study is completed, the investigators will offer a delayed intervention stage in which all clinics will receive online access to the MHMC tool.
- The choice of a comparison group was done carefully. Instead of an attention control group they are using a usual contraceptive care arm as a better reflection of clinical practice.
- Strong evidence of ability to recruit clinics to participate in the study.
- Power analysis is clear, attrition rate of 15% is reasonable, with good rationale provided for enrolling 14 clinics.
- Investigators are clear and specific about how data will be coded (e.g., in classifying contraceptive nonuse, the denominator will be adjusted if a patient reports trying to become pregnant or abstinence, method more effective will be chosen if >1 method reported, subclassifications for ratings for aim #2).
- Good description of the integration of quantitative and qualitative data.

Weaknesses

- Neither patients nor clinicians will be blinded to group assignment (which may be unavoidable).
- The choice of variables for purposeful sampling are not explained or referenced. No rationale is provided for seeking to balance on race, ethnicity, or setting.
- Thematic coding is not explained sufficiently. It is unclear how many people will be coding. No rationale or reference is provided for seeking a goal of 85-90% agreement in thematic analysis.

5. Environment:

Strengths

- UM is a world-class scientific environment. Facilities and resources are more than adequate to support the proposed study.
- Investigators have access to and relationships with clinics meeting the inclusion criteria to allow sample enrollment in a timely fashion.

Weaknesses

- None noted by reviewer.

Study Timeline:

Strengths

- Timeline is reasonable, without padding.

Weaknesses

- None noted by reviewer.

Protections for Human Subjects:

Acceptable Risks and/or Adequate Protections

Data and Safety Monitoring Plan (Applicable for Clinical Trials Only):

Acceptable

Inclusion Plans:

- Sex/Gender: Distribution justified scientifically
- Race/Ethnicity: Distribution justified scientifically
- For NIH-Defined Phase III trials, Plans for valid design and analysis: Scientifically acceptable
- Inclusion/Exclusion Based on Age: Distribution justified scientifically

Vertebrate Animals:

Not Applicable (No Vertebrate Animals)

Biohazards:

Not Applicable (No Biohazards)

Resource Sharing Plans:

Acceptable

Budget and Period of Support:

Budget Modifications Recommended (in amount/time)

Recommended budget modifications or possible overlap identified:

- The $20,000 remuneration to be given to each clinic seems high. The explanation of what the clinics will be doing is not sufficient to explain why this amount is budgeted.

CRITIQUE 3

Significance: 3
Investigator(s): 2
Innovation: 3
Approach: 3
Environment: 1

Overall Impact: This application addresses the significant clinical problem of contraceptive nonuse in women with chronic condition using a mobile app linked to a web based *My Health, My Choice (MHMC*) platform. The MHMC is based on a successful pilot study. The study proposes to a cluster randomized controlled trial guided by a theoretical framework. The investigators are a strong team, with 14 available clinic sites. The use of an interactive evidence-based guidelines decision tools is novel. Score driving weakness are clarity on recruitment criteria, consistency on data point collection, and rationale for post-interviews. Contraceptive decision-making is highly personal and often women with chronic conditions lack tools that guide decision about their contraceptive use.

1. Significance:

Strengths

- There has been a 40% increase in chronic conditions during pregnancy that contributes to the rise in maternal morbidity and mortality
- Women with chronic conditions are more likely to experience unplanned pregnancy and pregnancy-related complications than their peers without chronic conditions due to contraceptive nonuse.
- Contraceptive decision-making is a critical antecedent to contraceptive use, and a highly personal process.
- A significant gap is the lack of tools to address the unique contraceptive decisional needs of patients with chronic conditions.
- Long term impact will be to improve reproductive health outcomes and services through patient-centered contraceptive interventions.
- Based on Ottawa Decision Support Framework.

Weaknesses

- Women are provided with information before their clinical visit. A clear connection between the Ottawa Decision Support Framework and how those guide and supports an interactive discussion at point of care between clinician and women would strengthen the application.

2. Investigator(s):

Strengths

- The team includes a diverse array of expertise that favorably contribute to the project with history of collaboration.

- Team proficiencies include research experience in clinical expertise in supporting contraceptive planning.
- The primary investigator serves as a National Family Planning Fellowship Advisory Committee member
- The team designed the *My Health My Choice* decision tool during the PI's K23 award (5K23HD08474405).
- Established relationship with the American Academy of Family Physicians National Research Network which will facilitate recruitment of pregnant women with chronic conditions
- The team has experience and national expertise in mixed methods design

Weaknesses

- A gap is an expert in decision making or published application of the Ottawa Decision Support Framework

3. Innovation:

Strengths

- Uses an established clinical decision tool that has been user-tested, and has preliminary patient tested *My Health My Choice* data
- Provides clinician and patient with a range of chronic disease-relevant reproductive health and contraceptive education
- Point-of-care outpatient-based clinical support for clinicians that provides preventative patient-specific and harmonized with the CDC Contraceptive

Weaknesses

- Women nonuse of contraception in the pilot study was not significant, limiting the potential innovation

4. Approach:

Strengths

- Proof of concept of the use of decision-making tools to be used by clinicians and women and a point of interactive conversation.
- Strong partnership with American Academy of Family Physicians National Research Network which provides access to clinics for the RCT multi-site study
- Clinics will be selected based on clinical skill on a wide range of contraceptive methods (IUDs) and randomized to group
- Mid-level providers will be recruited to be recorded during their clinical discussion
- MEC guideless will be available to guide clinical discussion.
- Multi-step mixed methods RCT study with clear benchmarks
- Established mobile app platform with web-linked
- Entry data is deidentified

Weaknesses

- Pilot study intervention did not significantly decrease contraceptive nonuse
- Nine clinics have already volunteered to be part of the trial, but it unclear if they were assessed based on access to diversity, geographical location, or care setting (rural or urban)
- Recruitment of women occurs 2 – 3 weeks before a visit. Greater clarity to describe the inclusion criteria based on race/ethnicity and reason for clinic visit (routine care, contraceptive care, postpartum). Proposed enrollment table is 59% white with much lower enrollment planned for Blacks and Hispanic who are at higher risk for chronic diseases.
- Unclear if interviews will occur after saturation of ideas and themes
- Not available in Spanish

5. Environment:

Strengths

- Access to academic and to the network of clinics located in the U.S.

Weaknesses

- None noted by reviewer.

Study Timeline:

Strengths

- Reasonable timeline

Weaknesses

- None noted by reviewer.

Protections for Human Subjects:

Acceptable Risks and/or Adequate Protections

Data and Safety Monitoring Plan (Applicable for Clinical Trials Only):

Acceptable

Inclusion Plans:

- Sex/Gender: Distribution justified scientifically
- Race/Ethnicity: Distribution not justified scientifically
- For NIH-Defined Phase III trials, Plans for valid design and analysis:
- Inclusion/Exclusion Based on Age: Distribution justified scientifically
- The selection of clinics supports a largely White women

Vertebrate Animals:

Not Applicable (No Vertebrate Animals)

Biohazards:

Not Applicable (No Biohazards)

Resource Sharing Plans:

Not Applicable (No Relevant Resources)

Budget and Period of Support:

Budget Modifications Recommended (in amount/time)

Recommended budget modifications or possible overlap identified:

- Justification for the $20,000 stipend to the clinics

THE FOLLOWING SECTIONS WERE PREPARED BY THE SCIENTIFIC REVIEW OFFICER TO SUMMARIZE THE OUTCOME OF DISCUSSIONS OF THE REVIEW COMMITTEE, OR REVIEWERS' WRITTEN CRITIQUES, ON THE FOLLOWING ISSUES:

PROTECTION OF HUMAN SUBJECTS: ACCEPTABLE

INCLUSION OF WOMEN PLAN: ACCEPTABLE

INCLUSION OF MINORITIES PLAN: UNACCEPTABLE

- The selection of clinics supports a largely White women client population.

INCLUSION ACROSS THE LIFESPAN: ACCEPTABLE

COMMITTEE BUDGET RECOMMENDATIONS:

- The $20,000 remuneration to be given to each clinic seems high. The explanation of what the clinics will be doing is not sufficient to explain why this amount is budgeted.

Footnotes for 1 R01 HD110570-01; PI Name: WU, JUSTINE P

NIH has modified its policy regarding the receipt of resubmissions (amended applications).See Guide Notice NOT-OD-18-197 at https://grants.nih.gov/grants/guide/notice-files/NOT-OD-18-197.html. The impact/priority score is calculated after discussion of an application by averaging the overall scores (1-9) given by all voting reviewers on the committee and multiplying by 10. The criterion scores are submitted prior to the meeting by the individual reviewers assigned to an application, and are not discussed specifically at the review meeting or calculated into the overall impact score. Some applications also receive a percentile ranking. For details on the review process, see http://grants.nih.gov/grants/peer_review_process.htm#scoring.

MEETING ROSTER

Clinical Management in General Care Settings Study Section
Healthcare Delivery and Methodologies Integrated Review Group
CENTER FOR SCIENTIFIC REVIEW
CMGC
06/13/2022 - 06/14/2022

Notice of NIH Policy to All Applicants: Meeting rosters are provided for information purposes only. Applicant investigators and institutional officials must not communicate directly with study section members about an application before or after the review. Failure to observe this policy will create a serious breach of integrity in the peer review process, and may lead to actions outlined in NOT-OD-22-044 at https://grants.nih.gov/grants/guide/notice-files/NOT-OD-22-044.html, including removal of the application from immediate review.

CHAIRPERSON(S)

BADGER, TERRY A, RN, PHD
PROFESSOR
COLLEGE OF NURSING
UNIVERSITY OF ARIZONA
TUCSON, AZ 85721

MEMBERS

ALONSO, WINDY WILLIAMS, PHD *
ASSISTANT PROFESSOR
COLLEGE OF NURSING
UNIVERSITY OF NEBRASKA
OMAHA, NE 68198

APARASU, RAJENDER R, PHD
PROFESSOR AND CHAIR
DEPARTMENT OF PHARMACEUTICAL
HEALTH OUTCOMES AND POLICY
COLLEGE OF PHARMACY, TEXAS MEDICAL CENTER
UNIVERSITY OF HOUSTON
HOUSTON, TX 77204

ASHIDA, SATO, PHD *
ASSOCIATE PROFESSOR
DEPARTMENT OF COMMUNITY AND BEHAVIORAL HEALTH
CENTER ON AGING
UNIVERSITY OF IOWA
IOWA CITY, IA 52242

CAMPBELL, MARGARET LORENE, RN, PHD *
PROFESSOR
COLLEGE OF NURSING
WAYNE STATE UNIVERSITY
DETROIT, MI 48202

DEAN, GRACE E, PHD
PROFESSOR
SCHOOL OF NURSING
STATE OF UNIVERSITY NEW YORK
UNIVERSITY AT BUFFALO
BUFFALO, NY 14214

ESPINOZA, JUAN, MD *
ASSISTANT PROFESSOR
DEPARTMENT OF PEDIATRICS
CHILDREN'S HOSPITAL LOS ANGELES
UNIVERSITY OF SOUTHERN CALIFORNIA
LOS ANGELES, CA 90027

FAN, VINCENT S, MPH, MD
ASSOCIATE PROFESSOR
DEPARTMENT OF MEDICINE
UNIVERSITY OF WASHINGTON
SEATTLE, WA 98195

GANZ, PATRICIA A, MD *
PROFESSOR
DEPARTMENTS OF HEALTH SERVICES, MEDICINE,
HEMATOLOGY/ONCOLOGY
SCHOOLS OF MEDICINE AND PUBLIC HEALTH
UNIVERSITY OF CALIFORNIA, LOS ANGELES
LOS ANGELES, CA 90095

HURTADO DE MENDOZA CASAUS, ALEJANDRA, PHD *
ASSISTANT PROFESSOR
POPULATION SCIENCES
GEORGETOWN UNIVERSITY
WASHINGTON, DC 20057

KEIM-MALPASS, JESSICA, PHD *
ASSOCIATE PROFESSOR
SCHOOL OF NURSING
UNIVERSITY OF VIRGINIA
CHARLOTTESVILLE, VA 22903

KEMPER, ALEX R, MPH, MD
PROFESSOR
DEPARTMENT OF PEDIATRICS
NATIONWIDE CHILDREN'S HOSPITAL
THE OHIO STATE UNIVERSITY
COLUMBUS, OH 43205

KRANS, ELIZABETH E, MD
ASSOCIATE PROFESSOR
DEPARTMENT OF OBSTETRICS, GYNECOLOGY
AND REPRODUCTIVE SCIENCES
MAGEE-WOMEN'S HOSPITAL
UNIVERSITY OF PITTSBURGH MEDICAL CENTER
PITTSBURGH, PA 15213

LEVIN, JENNIFER BETH, PHD
ASSOCIATE PROFESSOR
DEPARTMENT OF PSYCHIATRY
SCHOOL OF MEDICINE
CASE WESTERN RESERVE UNIVERSITY
CLEVELAND, OH 44106

LUCAS, RUTH F, PHD *
ASSISTANT PROFESSOR
SCHOOL OF NURSING
UNIVERSITY OF CONNECTICUT
WILLINGTON, CT 06279

LUM, HILLARY, MD, PHD *
ASSOCIATE PROFESSOR
DEPARTMENT OF MEDICINE-GERIATRICS
SCHOOL OF MEDICINE
UNIVERSITY OF COLORADO DENVER
AURORA, CO 80045

MAGWOOD, GAYENELL SMITH, RN, PHD
PROFESSOR
DEPARTMENT OF NURSING
COLLEGE OF NURSING
MEDICAL UNIVERSITY OF SOUTH CAROLINA
CHARLESTON, SC 29425

NELSON, LONNIE A, PHD
ASSOCIATE PROFESSOR
COLLEGE OF NURSING
WASHINGTON STATE UNIVERSITY
SPOKANE, WA 99202

NELSON, LYNDSAY ANN, PHD *
ASSISTANT PROFESSOR
DEPARTMENT OF GENERAL INTERNAL MEDICINE
AND PUBLIC HEALTH
VANDERBILT UNIVERISITY
NASHVILLE, TN 37204

PIATT, GRETCHEN A, MPH, PHD
ASSOCIATE PROFESSOR
DEPARTMENT OF LEARNING HEALTH SCIENCES
UNIVERSITY OF MICHIGAN
ANN ARBOR, MI 48109

PICKERING, CAROLYN E ZIMINSKI, RN, PHD
ASSOCIATE PROFESSOR
SCHOOL OF NURSING
UNIVERSITY OF ALABAMA, BIRMINGHAM
BIRMINGHAM, AL 35226

QUINONES, ANA ROMAN, PHD
ASSOCIATE PROFESSOR
DEPARTMENT OF FAMILY MEDICINE
SCHOOL OF MEDICINE
OREGON HEALTH AND SCIENCE UNIVERSITY
PORTLAND, OR 97239

REED, MONIQUE A, PHD *
ASSOCIATE PROFESSOR
DEPARTMENT OF COMMUNITY, SYSTEMS
AND MENTAL HEALTH NURSING
COLLEGE OF NURSING
RUSH UNIVERSITY
CHICAGO, IL 60653

RIEGEL, BARBARA J, DNSC, RN *
PROFESSOR
SCHOOL OF NURSING
UNIVERSITY OF PENNSYLVANIA
PHILADELPHIA, PA 19104-4217

SUN, VIRGINIA CHIH-YI, RN, PHD *
ASSOCIATE PROFESSOR
DEPARTMENT OF POPULATION SCIENCES EDUCATION
CITY OF HOPE
DUARTE, CA 91010

TORKE, ALEXIA M, MD
PROFESSOR
CENTER OF AGING RESEARCH
INDIANA UNIVERSITY
INDIANAPOLIS, IN 46202

VRANCEANU, ANA-MARIA, PHD *
ASSOCIATE PROFESSOR
DEPARTMENT OF PSYCHOLOGY
HARVARD MEDICAL SCHOOL
BOSTON, MA 02476

WALKER, REBEKAH J, PHD *
ASSOCIATE PROFESSOR
DEPARTMENT OF MEDICINE
MEDICAL COLLEGE OF WISCONSIN
MILWAUKEE, WI 53226

WELLS, KRISTEN JENNIFER, MPH, PHD
PROFESSOR
DEPARTMENT OF PSYCHOLOGY
SAN DIEGO STATE UNIVERSITY
SAN DIEGO, CA 92120

WILLIAMS, KRISTINE N, PHD
PROFESSOR
SCHOOL OF NURSING
UNIVERSITY OF KANSAS MEDICAL CENTER
KANSAS CITY, KS 66160

YEE, LYNN M, MD *
ASSISTANT PROFESSOR
DEPARTMENT OF OBSTETRICS AND GYNECOLOGY
FEINBERG SCHOOL OF MEDICINE
NORTHWESTERN UNIVERSITY
CHICAGO, IL 60611

MAIL REVIEWER(S)

ZHU, HONG, PHD
ASSOCIATE PROFESSOR
DEPARTMENT OF POPULATION AND DATA SCIENCES
UNIVERSITY OF TEXAS SOUTHWESTERN MEDICAL CENTER
DALLAS, TX 75390

SCIENTIFIC REVIEW OFFICER

FORDYCE, LAUREN, PHD
SCIENTIFIC REVIEW OFFICER
CENTER FOR SCIENTIFIC REVIEW
NATIONAL INSTITUTES OF HEALTH
BETHESDA, MD 20892

EXTRAMURAL SUPPORT ASSISTANT

MACPHERSON, SETH LOPAKI
EXTRAMURAL SUPPORT ASSISTANT
CENTER FOR SCIENTIFIC REVIEW
NATIONAL INSTITUTES OF HEALTH
BETHESDA, MD 20892

* Temporary Member. For grant applications, temporary members may participate in the entire meeting or may review only selected applications as needed.

Consultants are required to absent themselves from the room during the review of any application if their presence would constitute or appear to constitute a conflict of interest.
